# Identifying N-linked glycan moiety and motifs in the cysteine-rich domain critical for N-glycosylation and intracellular trafficking of SR-AI and MARCO

**DOI:** 10.1186/s12929-016-0244-5

**Published:** 2016-02-18

**Authors:** Huey-Jen Tsay, Yung-Cheng Huang, Yi-Jen Chen, Yun-Hao Lee, Shu-Meng Hsu, Keng-Chang Tsai, Cheng-Ning Yang, Fong-Lee Huang, Feng-Shiun Shie, Lin-Chien Lee, Young-Ji Shiao

**Affiliations:** Institute of Neuroscience, Brain Research Center, National Yang-Ming University, Taipei, 11221 Taiwan R.O.C; Department of Physical Medicine and Rehabilitation, Cheng Hsin General Hospital, Taipei, 112 Taiwan R.O.C; Division of Chinese Materia Medica Development, National Research Institute of Chinese Medicine, Taipei, 11221 Taiwan R.O.C; Institute of Anatomy and Cell Biology, National Yang-Ming University, Taipei, 11221 Taiwan R.O.C; Center for Neuropsychiatric Research, National Health Research Institutes, No. 35 Keyan Road, Zhunan Town, Miaoli County, 350 Taiwan R.O.C; Department of Physical Medicine and Rehabilitation, Cheng Hsin General Hospital, Taipei, 11220 Taiwan R.O.C; Division of Basic Chinese Medicine, National Research Institute of Chinese Medicine, Taipei, 11221 Taiwan R.O.C; Institute of Biopharmaceutical Science, National Yang-Ming University, Taipei, 11221 Taiwan R.O.C

**Keywords:** Scavenger receptor A, MARCO, SRCR domain, N-glycosylation, Alzheimer’s disease, oligomeric β-amyloid

## Abstract

**Background:**

The accumulation of soluble oligomeric amyloid-β peptide (oAβ) proceeding the formation of senile plaques contributes to synaptic and memory deficits in Alzheimer’s disease. Our previous studies have indentified scavenger receptor A (SR-A), especially SR-A type I (SR-AI), as prominent scavenger receptors on mediating oAβ clearance by microglia while glycan moiety and scavenger receptor cysteine-rich (SRCR) domain may play the critical role. Macrophage receptor with collagenous structure (MARCO), another member of class A superfamily with a highly conserved SRCR domain, may also play the similar role on oAβ internalization. However, the role of N-glycosylation and SRCR domain of SR-AI and MARCO on oAβ internalization remains unclear.

**Result:**

We found that oAβ internalization was diminished in the cells expressing SR-AI harboring mutations of dual N-glycosylation sites (i.e. N120Q-N143Q and N143Q-N184Q) while they were normally surface targeted. Normal oAβ internalization was observed in 10 SR-AI-SRCR and 4 MARCO-SRCR surface targeted mutants. Alternatively, the SRCR mutants at β-sheet and α-helix and on disulfide bone formation obstructed receptor’s N-glycosylation and surface targeting.

**Conclusion:**

Our study reveals that N-glycan moiety is more critical than SRCR domain for SR-A-mediated oAβ internalization.

**Electronic supplementary material:**

The online version of this article (doi:10.1186/s12929-016-0244-5) contains supplementary material, which is available to authorized users.

## Background

Activated scavenger receptor A (SR-A) promotes glial internalization of Amyloid-β peptide (Aβ), which is a key histopathological characteristic of Alzheimer’s disease (AD) [1, 2]. Previously, we identified SR-A as the prominent subtype of scavenger receptor mediating oligomeric Aβ (oAβ) internalization in microglia, and revealed that the cysteine-rich (SRCR) domain of SR-A type I (SR-AI) may be the critical domain on modulating the efficacy of surface targeting and mediating oAβ internalization [3, 4].

**Fig. 1:**
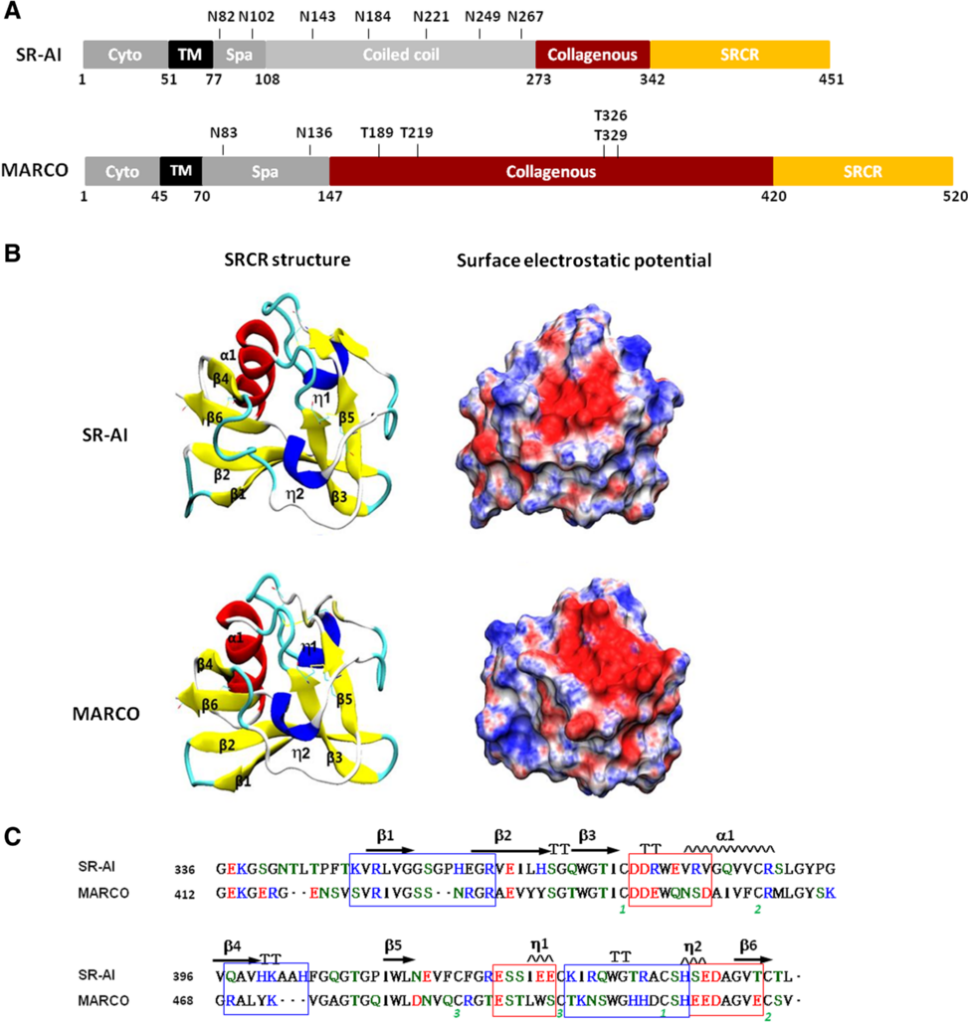
Modeled structure of the SRCR domain of human SR-AI and MACRO. **a** Schematic illustration of human MCRCO and SR-AI. The MARCO domains: cytoplamic domain (cyto), transmembrane domain (TM), spacer domain (Spa), coiled coil domain (coiled coil), triple-helical collagenous domain (collagenous), and cysteine-rich C-terminal domain (SRCR). The predicted N-glycosylation sides and amino acid sequence number was marked above and below the schematic illustration, respectively. **b** The structure of the SRCR domain of SR-AI and MACRO is composed of six β-strands (β1-6, *yellow*), one α-helix (α1, *red*), two 3_10_ helices (η1, η2, *blue*), turns (*indigo*), and loops (*gray*). Disulfide bonds are shown in orange. The conformation folds with three β-strands (β1, β2, β3) at the N-terminal region followed by a short turn, α-helix (α1), and β4. Then, there are three long loops, located between β4 and β5, between β5 and η1, and between η1 and η2, ending with β6. Surface electrostatic potential representation of the SRCR domain of SR-AI and MACRO with basic (*blue*) and acidic (*red*) clusters. **c** Sequence alignment of human SR-AI and MACRO were displayed using ClustalW2 and ESPrip. The β-sheets are shown as arrows, and helixes are shown as a saw tooth pattern. Turns are marked as TT. The three pairs of disulfide bonds are labeled as numbers in *green*

SR-AI contains six domains: a cytoplasmic domain, a transmembrane domain, a spacer region, an α-helical coiled-coil domain, a collagenous domain, and a C-terminal SRCR domain [5]. The cytoplasmic domain of SR-A has been identified to involve in cell adhesion and receptor internalization [6]. The critical amino acids in cytoplasmic domain involving SR-A surface targeting and interaction with signaling molecules have been identified [7, 8]. Seven residues in the α-helical coiled-coil domain mediate the formation of the trimeric coiled-coil structure [9]. The collagenous domain mediates binding to the extracellular matrix [10], and point mutations in the positively charged lysine clusters in the SR-A type II collagenous domain have been shown to decrease acetylated-LDL (AcLDL) binding activity [11]. Macrophage receptor with collagenous structure (MARCO), another SRCR-containing group A receptor, shows the same overall domain structure as the SR-AI but differs in having a longer extracellular domain and completely lacking an α-helical coiled-coil domain [12]. The schematic representation of human SR-AI and MARCO were shown in Fig. 1a. The modeled structures and surface electrostatic potential of the SRCR domain of human SR-AI and MARCO were shown in Fig. 1b. The sequence of the SRCR domain is highly conserved between human SR-AI and human MARCO ([13], Fig. 1c). Examination of the crystal structure of the mouse MARCO SRCR domain revealed that the monomeric recombinant SRCR domain possess a compact and globular conformation, which was identified as the binding domain for bacteria, AcLDL, and the extracellular matrix [14, 15].

Protein glycosylation have important biological functions in protein folding and stability, cell adhesion, receptor targeting and ligand binding [16]. Previous study of SR-A showed that murine SR-A-mediated AcLDL uptake was not significantly affected even though the N-linked glycosylation of SR-A is prevented [17]. The surface-targeted SR-A remains at high mannose status, suggested that the absence of complex N-glycan does not impair the trafficking and AcLDL uptake of SR-A. However, the molecular mechanisms underlying SR-AI activity and the relationship of the structure of SR-AI to its functions are not well-understood. Previous studies have conducted several studies involving the generation and characterization of SR-BI mutants [18]. Some of these studies have shown that the large, extracellular loop of SR-BI, which is glycosylated, plays a critical role in mediating not only ligand binding but also the selective lipid uptake step [18]. The functional role of the extensive *N*-linked glycosylation of SR-BI has also been explored [19].

Previous studies have demonstrated that microglia take up and degrade soluble and fibrillar Aβ in different ways. Soluble Aβ is internalized into microglia through constitutive, non-saturable, and fluid phase macropinocytosis, and the internalized soluble Aβ is rapidly delivered to the lysosomes by the late endolytic pathway [20]. Fibrillar Aβ interacts with the cell surface innate immune receptor complex and then stimulates phagocytosis and degradation by microglia [21–26]. The putative role of MARCO has been discussed for internalization and Aβ1-42-mediated microglia activation [22, 27]. As some studies have failed to confirm the ability of MARCO to mediate the proinflammatory activity of Aβ1-42 in mononuclear phagocytes further studies must clarify such interaction [28].

In the present study, we reveal that N-glycan moiety is more critical than SRCR domain for oAβ internalization mediated by SR-AI. Our results provide insight into the critical role of N-glycosylation of SR-AI, which is a prerequisite for the uptake of oAβ by microglia in the initiation stage of AD.

## Methods

### Reagents

Fluorescein amidite (FAM)-labeled Aβ 1–42 were purchased from Biopeptide (San Diego, CA). Antibodies against MARCO were purchased from Santa Cruz Biotechnology, Inc. (Santa Cruz, CA). Rabbit anti-transferrin receptor (TfR) antibody and Lipofectamine 2000 were purchased from Invitrogen (Carlsbad, CA). Mouse anti-β-actin antibody was purchased from Sigma-Aldrich (St. Louis, MO). Sulfo-NHS-SS-biotin and NeutrAvidin were purchased from Pierce (Rockford, IL). Peptide N-glycosidase (PNGase F) and endoglycosidase (Endo H) were purchased from New England BioLabs (Ipswich, MA).

### Modeling

The crystal structure of mouse MARCO (Protein Data Bank entry 2oy3) [15] was used as a template. A homology modeling method was used to construct the tertiary structure of the SRCR domain of SR-AI [29]. The modeled structure was then optimized, followed by molecular dynamics simulations using the GROMACS (GROningen MAchine for Chemical Simulations) program version 4.5.1 with the AMBER99SB force field [30].

### Cell culture

COS-7 (African green monkey kidney fibroblast-like cell line) cells were maintained in DMEM containing 10 % heat-inactivated fetal bovine serum (FBS), 100 U/mL penicillin, 100 μg/mL streptomycin, and 2 mM L-glutamine at 37 °C in a 5 % CO_2_ humidified atmosphere. The human embryonic kidney cell line 293 T (HEK293T) were cultured in DMEM supplemented with 10 % FBS, 100 U/ml penicillin, 100 μg/ml streptomycin, and 2 mM L-glutamine in a humidified 5 % CO2 atmosphere at 37 °C.

### Plasmid construction and transfection

Human SR-AI cDNA was provided by Dr. Qi Chen (Nanjing Medical University, Nanjing, China). The vector, pcDNA 3.1(+) (Invitrogen, USA) was used to insert full length cDNAs of human SR-AI [4] and MARCO. The sequences of primers and ligation sites used to construct SR-AI variants are shown in Additional file 1: Table S1. Site-directed mutagenesis was performed by using QuickChange II Site-Directed Mutagenesis Kit (Stratagene, La Jolla, CA) according to the manufacturer’s instructions. COS-7 cells (3 × 10^5^) were transfected with 2 μg SR-AI or variants per well in 6-well plates using Lipofectamine 2000 according to manufacturer instructions. After 24 h, cells were subjected to ligand binding, surface protein biotinylation, and immunocytochemical analyses. The full-length of human MARCO was provide by Genomic Research Center, National Yang-Ming University (Taipei, Taiwan). The sequences of primers and PCR condition used to construct MARCO variants including deletion and point mutants were listed in Additional file 1: Table S2. Site-directed mutagenesis was performed by using QuickChange II Site-Directed Mutagenesis Kit (Stratagene, La Jolla, CA) according to the manufacturer’s instructions. HEK293T cells (3 × 10^5^) were transfected with 2 μg MARCO or variants per well in 6-well plates using Lipofectamine 2000 according to manufacturer instructions. After 24 to 48 h, cells were subjected to ligand binding, surface protein biotinylation, and immunocytochemical analyses.

### Immunocyochemistry and live immunostaining

Live cells were incubated with rabbit anti-human SR-A antibody at 1:500 dilution (catalog number sc-20660, Santa Cruz Biotechnology) without permeabilization. Cells were incubated with secondary antibody conjugated to Alexa Fluor 488 to detect surface-targeted SR-A. To detect cytosolic SR-A, permeabilized mouse primary microglia were then incubated with rat anti-mouse SR-A (catalog number MCA1322EL, AbD Serotec). Permeabilized human macrophage and transfected COS-7 cells were incubated with rabbit anti-human SR-A, followed by incubation with secondary antibody conjugated to Alexa Fluor 594. Coverslips were mounted with Vitashield (catalog number H-1200, Vector Laboratories) and images were taken using a confocal microscope (Olympus, FV-1000 and FV-10i). The experiments were repeated at least three times.

To detect the surface-targeted MARCO, live cells were incubated with anti-MARCO antibody (H190, 1:100) at 4 °C. Cells were then fixed and followed by incubation with secondary antibody conjugated to AlexaFluor 488 or 594 secondary antibodies. The coverslips were mounted in Vitashield (Vector Laboratories, Burlingame, CA) and observed using a Zeiss confocal microscope (LSM780). The experiments were repeated at least three times.

### Western blot analysis

Cells were lysed in lysis buffer (50 mM Tris, pH 7.4, 150 mM NaCl, 1 % NP-40, 0.25 % Sodium deoxycholate and protease inhibitor cocktail. After electrophoresis, proteins were transferred onto PVDF membranes (NEN Life Science Products, Boston, MA). After blocking, the membranes were incubated overnight at 4 °C with primary antibodies for MARCO at a 1:1,000 dilution and β-actin at a 1:5,000 dilution. After incubation with secondary antibody (goat anti-mouse antibody or goat anti-rabbit antibody conjugated to horseradish peroxidase), the immune complexes were detected using an enhanced chemiluminescence kit (NEN Life Science Products, Boston, MA). The band intensities were quantified by densitometry. More than three independent experiments were performed.

### Surface biotinylation assay

Surface proteins were labeled with Sulfo-NHS-SS-biotin in PBS-MC according to the manufacturer’s instructions (Pierce, Rockford, IL). Briefly, cells were incubated with 0.5 mg/ml of membrane-impermeable Sulfo-NHS-SS-biotin in PBS-MC on ice for 30 min. Unbound biotin was quenched by Tris buffer (25 mM Tris-HCl [pH 8.0], 133 mM NaCl, and 10 mM KCl) for 10 min on ice. Cells were lysed with lysis buffer (50 mM Tris, pH 7.4, 150 mM NaCl, 1 % NP-40, 0.25 % Sodium deoxycholate and protease inhibitor cocktail. Biotinylated proteins (400 mg) were precipitated using 50 ml Neutravidin beads and eluted by boiling in lysis buffer followed by immunoblotting for individual proteins.

### Deglycosylation

Cleavage of the *N*-glycan was performed using PNGase Fand Endo H as described [4]. Briefly, total cell lysates and biotinylated surface proteins were denatured in denaturing buffer at 95 °C for 10 min. The protein mixtures were incubated with PNGase F (500 units) or Endo H (1000 units) for 18 hr at 37 °C. The protein was boiled for 10 min at 95 °C subjected to Western blot analysis after adding an equal volume of 2× laemmli SDS sample buffer (BioRad).

### Internalization of oligomeric Aβ

FAM-labeled oligomeric Aβ (oAβ**)** was prepared from monomer Aβ_1-42_, synthesized by Biopeptide, as previously described [3, 4]. The oligomeric Aβ42 preparation preformed in our laboratory has been characterized by atomic force microscope and Sedimentation velocity analysis [3]. The composition of oligomeric Aβ42 in the grown medium remains stable as analyzed by Western blot analysis. SR-A cells were incubated with 1 μM FAM-oAβ in serum-free DMEM at 37 °C for 1 h. Then, cells were immunostained with the anti-SR-A antibody. Images were taken using a confocal microscope (Olympus, FV-1000 and FV-10i). The experiments were repeated at least three times. The fluorescence intensities of more than 100 SR-A-positive cells in five random fields were analyzed using the MetaMorph software (ver. 7.1; Molecular Devices).

### Statistical analysis

Data were expressed as mean ± SEM and the differences between groups were considered significant when the values of *p* < 0.05. The comparison between groups was analyzed by one-way analysis of variance (ANOVA) followed by Tukey’s Honestly Significant Differences Multiple Comparison Test as *post-hoc* analysis using GraphPad Prism 4 (GraphPad Software Inc., San Diego, CA, USA).

## Results

### Identification of *N*-linked glycosylation sites on SR-AI and MARCO by mutagenesis

**Fig. 2:**
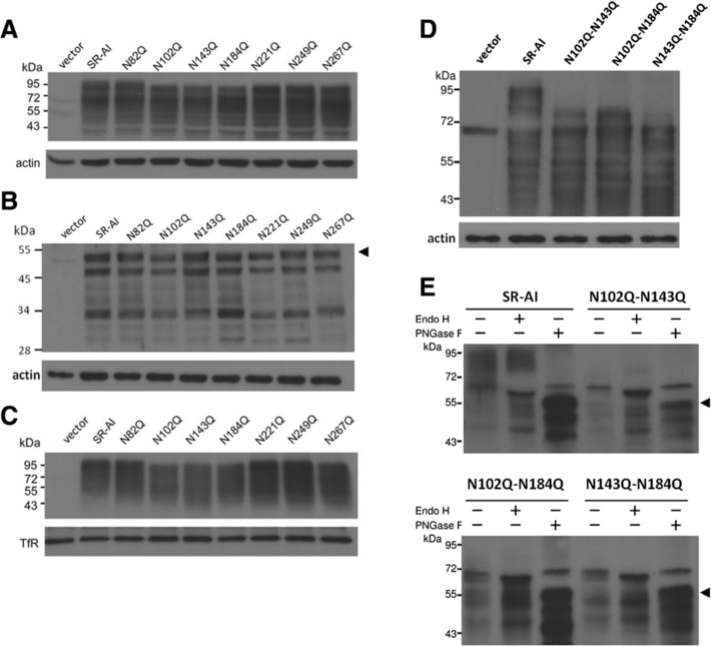
Identification of the potential N-glycosylation sites of SR-AI. **a** and **b** COS 7 cells were transfected with human SR-AI, the single mutation of asparagine to glutamine at residues 82, 102, 143, 184, 221, 249 and 267. Western blot analysis was performed on the total cell lysates before (**a**) and after (**b**) treated by PNGase F, and avidin pull-down of biotinylated cell lysates (**c**). **d** and **e** COS-7 cells were transfected with hSR-AI and the double mutation of asparagine (N) to glutamine (Q) residues at 102-143, 102-184 and 143-184. Western blot analysis was performed on the total cell lysates (**d**) and the PNGase F or Endo H treated cell lysates (**e**). The result was repeated for three times (N = 3) and the representative blot was shown

Seven potential *N*-linked glycosylation sites were predicted in human SR-AI as those predicted by Asn-*X*-Ser/Thr sequence in extracellular domain of SR-BI [17, 19]; these include the asparagines at positions: 82, 102, 143, 184, 221, 249, and 267. To determine their roles in structure and function of SR-AI, we mutated these sites individually or in combination by replacing asparagine (N) with glutamine (Q) (Fig. 1a and Additional file 1: Table S1). Their glycosylation and surface targeting of the mutants were then analyzed (Fig. 2). The results showed that the largest molecular weight of the SR-AI mutated at N102Q, N143Q, and N184Q were lower (~80 kDa) than that of wild-type SR-AI (~95 kDa) in both total cell lysates (Fig. 2a) and membrane portion (Fig. 2c), but not in the PNGase F cleaved total cell lysates (Fig. 2b). To go a step further, the effects of double mutations at N102Q, N143Q, and N184Q were examined. Western blot analysis showed that the largest molecular weight of the SR-AI mutated at N102Q-N143Q, N102Q-N184Q and N143Q-N184Q were smaller (~72 kDa) than single mutant (~80 kDa) and wild-type (~95 kDa) in total cell lysates (Fig. 2d). After PNGase F cleavage, the largest molecular weight of N102Q-N143Q, N102Q-N184Q, N143Q-N184Q and wild type were equally reduced to 55 kDa (Fig. 2e). After Endo H cleavage, the molecular weight range of wild type SR-AI falls into two parts with 50-60 kDa and 72-95 kDa, respectively. However, SR-A with higher molecular weight was absent in the mutant at N102Q-N143Q, N102Q-N184Q and N143Q-N184Q, suggesting that the N-glycan at 102, 143 and 184 are larger than the other N-glycosylation sites (Fig. 2d).

### oAβ internalization was down-regulated in the cells expressing SR-AI mutated at N102Q-N143Q and N143Q-N184Q

**Fig. 3:**
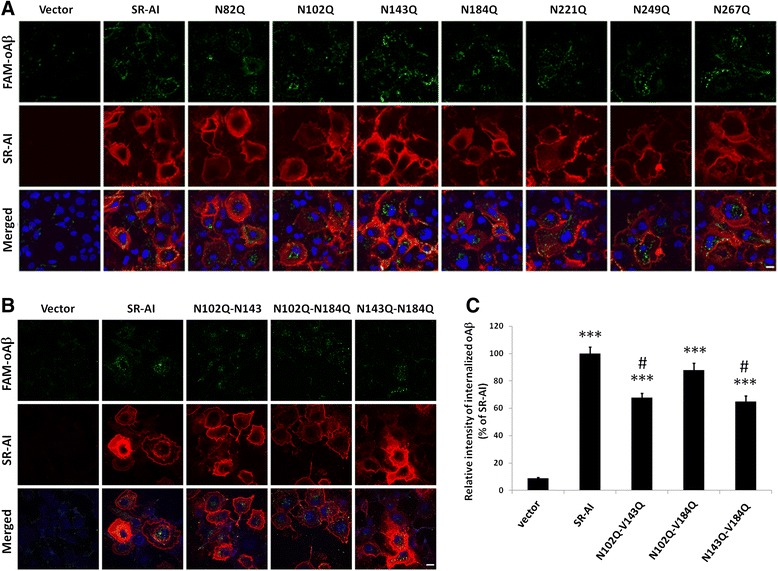
The glycosylation at N102-N143 and N143-N184 of SR-AI were important for oAβ internalization. COS-7 cells were transfected with human SR-AI, the single mutation of asparagine residues at 82, 102, 143, 184, 221, 249 and 267 to glutamine or the double mutation of asparagine residues at 102-143, 102-184 and 143-184 to glutamine. Cells were incubated with FAM-labeled oAβ. **a** and **b** Representative confocal images of surface-targeted SR-AI (*red*) and internalized FAM-oAβ (*green*) of the cells tranfected with SR-AI-single mutant (**a**) and double mutant (**b**). Nuclei were stained with Hoechst 33258 (*blue*). Scale bar, 10 μm. The result was repeated for three times (N = 3). **c** Relative fluorescence intensities of internalized oAβ were quantified in more than 100 SR-A-positive cells using MetaMorph software. Bars indicate mean ± SEM of at least three independent experiments. The data are presented as the percentage relative to the wild type SR-AI transfected cells. Significant differences between vector and wild type SR-AI transfected cells are indicated by ***, P < 0.001. Significant differences between wild type SR-AI transfected cells and the mutant transfected cells are indicated by #, P < 0.05

To determine the surface targeting and oAβ internalization, live immunostaining of SR-AI and internalization of FAM-labeled oAβ was performed (Fig. 3). The live immunostaining showed that all mutated SR-AI were surface targeted (Figs. 2b and 3a). We also found that the oAβ internalization was significantly reduced in the cells expressing SR-AI mutated at N102Q-N143Q and N143Q-N184Q (Fig. 3b), suggesting that the N-glycosylation at N102-N143 and N143-N184 sites are critical for SR-AI to internalized oAβ (Table 1).

**Table 1:** Summary of the surface targeting and oAβ internalization of mutants in N-glycosylation sites of human SR-AI

Mutants	Complex glycan structure	Surface targeting	oAβ internalization
N82Q	✚	✚	✚
N102Q	**↓**	✚	✚
N143Q	**↓**	✚	✚
N184Q	**↓**	✚	✚
N221Q	✚	✚	✚
N249Q	✚	✚	✚
N267Q	✚	✚	✚
N102Q- N143Q	━	✚	**↓**
N102Q- N184Q	━	✚	✚
N143Q- N184Q	━	✚	**↓**

Complex glycan structure is able to be cleavage by PNGase F but not by Endo H; Hybrid and high mannose type glycans is able to be cleavage by both PNGase F and Endo H

✚, mutants are glycosylated in complex structure, surface-targeted or internalize normal amount of oAβ

━, mutants are not glycosylated in complex structure

**↓**, mutants are less glycosylated in complex structure or internalize less amount oAβ

### Critical residues in SRCR domain of SR-AI for the surface targeting and N-glycosylation were identified by point mutation

**Fig. 4:**
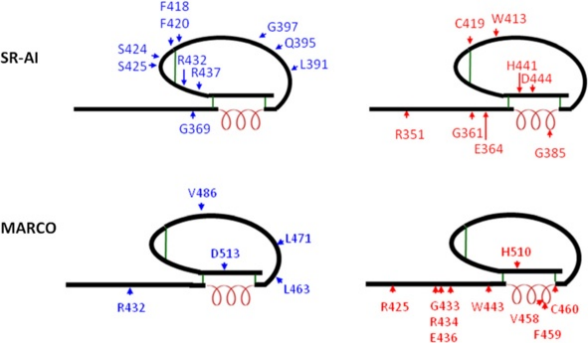
Schematic representation shows the SRCR mutations in SR-AI and MARCO. The mutations sites at loop structure are shown in blue and those at α-helix, β-sheet, η-helix and disulfide structure are shown in *red*. The *black* line indicates the sequence of SRCR; α-helix is indicated as red spring, and disulfide bonds are shown as green line

To determine the critical residues of the SR-AI SRCR domain for N-glycosylation and surface targeting of SR-AI, and SR-AI-mediated oAβ internalization, the positively charged (R), negatively charged (E), polar uncharged (Q and S) and hydrophobic (G, V, W and F) amino acids in β-sheet 1 (R351), β-sheet 2 (E360, R362 and E364), β-sheet 3 (W371), α-helix (Q385), β-sheet 4 (V399), β-sheet 5 (W413), disulfide bond (C419), 3_10_ helix η2 to β-sheet 6 (D444) and loop (L391, G395-Q397, F418, F420, S424-S425, R432 and R437) were site-directed mutated to hydrophobic alanine (A) (Fig. 4 and Additional file 1: Table S2). Moreover, amino acid residuals in β-sheet 2 (G361), turn (G369) and 3_10_ helix η2 (H441) were also site-directed mutated to P, S, and R, respectively (Fig. 4 and Additional file 1: Table S2).

**Fig. 5:**
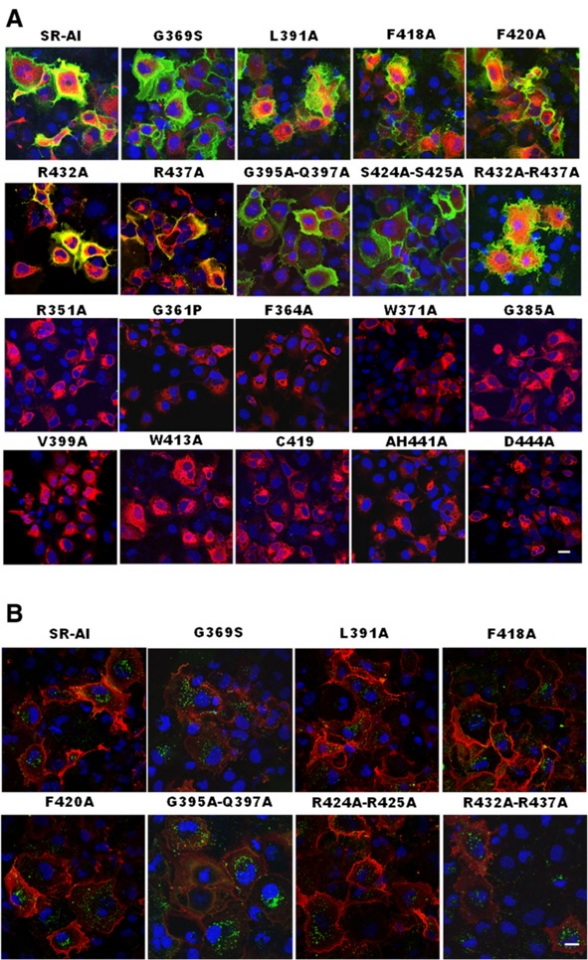
Critical residues in SRCR domain of human SR-AI for the surface targeting and N-glycosylation were identified by point mutation. COS-7 cells were transfected with wild type SR-AI and SR-AI variants. **a** Surface-targeted SR-AI and its variants were detected by live immunostaining (*green*). Cytosolic SR-A and its variants were detected by immunocytochemistry (red). Nuclei were counterstained with Hoechst 33258 (*blue*). **b** Cells were incubated with FAM-labeled oAβ. Representative merged confocal images of surface-targeted SR-AI (red) and internalized FAM-labeled oAβ (*green*) were shown. Nuclei were counterstained with Hoechst 33258 (*blue*). Scale bar, 10 μm. The result was repeated for four times (N = 4) and the representative image was shown

The immunocytochemistry analysis showed that the SR-AI mutated at G361P, E364A, H441R, G385A, W413A, C419A, H441A and D444A were expressed in cytoplasm only (red) and the SR-AI mutated at G369S, L391A, F418A, F420A, R432A, R437A, G395A-Q397A, S424A-S425A and R432A-R437A were expressed in both cytoplasm and cell surface (green and yellow) (Fig. 5a). For detecting the effect of mutation on oAβ internalization, the cells expressing the SR-AI mutated at G369S, L391A, F418A, F420A, R432A, R437A, G395A-Q397A, S424A-S425A and R432A-R437A were incubated with FAM-oAβ and internalization were detected. The result showed that the green punta signals were detected in all of detected cells (Fig. 5b).

**Fig. 6:**
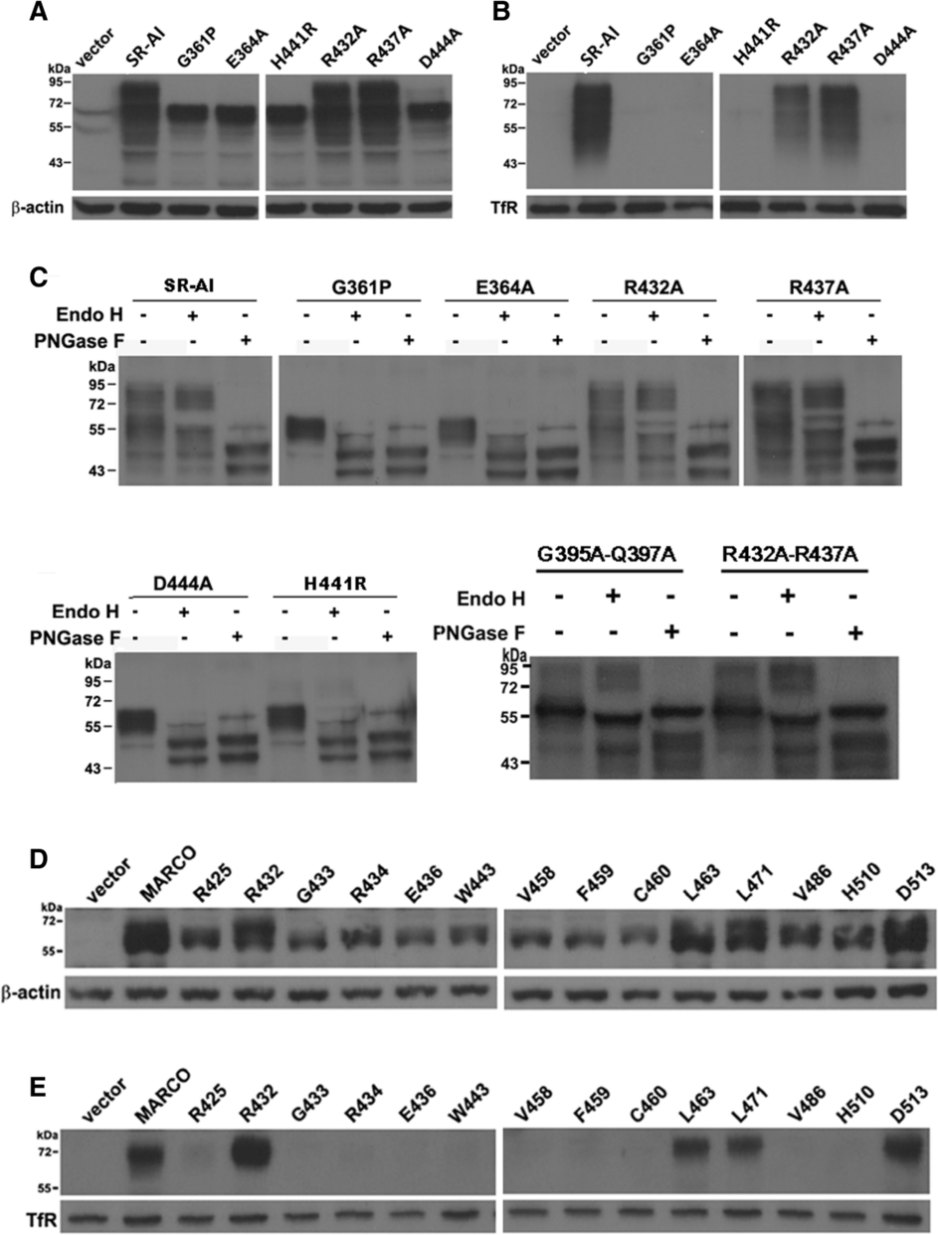
Critical residues in the SRCR domain for the surface targeting and N-glycosylation of SR-AI and MARCO. **a**-**c** SR-AI variant G361P, E364A, S369S, L391A, F418A, F420A, G395A-Q397A, S424A-S425A, R432A, R437A, R4321A-R437A, H441R, and D444A were created by site-directed mutagenesis. Total cell lysates (A) and avidin pull-down of biotinylated lysates (B) were subjected to Western blot analysis. Western blot analysis of total cell lysates after PNGase F or Endo H cleavage (C). **d**-**e**, HEK293 cells were transfected with MARCO and variant R425A, R432A, G433A, R434A, E436A, W443A, V458A, F459A, C463A, L471A, V486A, H510A and D513A were created by site-directed mutagenesis. Total cell lysates (D) and avidin pull-down of biotinylated lysates (E) were subjected to Western blot analysis. The result was repeated for four times (N = 4) and the representative blot was shown

Two out of 11 surface targeted mutants (i.e. R432A and R437A) and 4 out of 10 intracellular retained mutants (i.e. G361P, E364A, H441R, and D444A) were selected for Western blots analysis. The result showed that the electrophoretic mobility range of the mutant at R432A and R437A were similar to wild type SR-AI (from 45 to 100 kDa), suggesting that they were glycosylated to a similar extent. Alternatively, the range of electrophoretic mobility of the mutant at G361P, E364A, H441R, and D444A were narrow down (from 55 to 72 kDa), suggesting that the N-glycosylation of these mutants may be held at a premature stage (Fig. 6a). Furthermore, the surface biotinylation assay showed that only R432A and R437A were surface-targeted (Fig. 6b).

The N-glycosylation status of wild type SR-AI and mutant G361P, E364A, R432A, R437A, H441R, D444A, G395A-Q397A and R432A-R437A were assessed by Western blotting after the cell extracts were incubated with Endo H and PNGase F (Fig. 6c). The result showed that Wild type SR-AI and mutant R432A, R437A, G395A-Q397A and R432A-R437A were sensitive to PNGase F but not to Endo H, indicating the N-linked complex glycans. Alternatively, mutant G361P, E364A, H441R and D444A were sensitive to both PNGase F and Endo H, suggesting the high mannose- or hybrid-type glycans (Fig. 6c). These results showed that all mutation in the domains with secondary structure retain receptor intracellularly and abolished N-glycosylation of SR-AI (Table 2).

**Table 2:** Summary of the surface targeting and oAβ internalization of mutants in SRCR domain of human SR-AI

Mutants	Mutation position	Surface targeting	oAβ internalization
R351A	β1	━	━
E360A	β2	━	━
G361P	β2	━	━
R362A	β2	━	━
E364D	β2	━	━
G369S	Turn	✚	✚
W371A	β3	━	━
Q385A	α1	━	━
L391A	Loop	✚	✚
G395A-Q397A	loop-β4	✚	✚
V399A	β4	━	━
W413A	β5	━	━
F418A	loop	✚	✚
C419A	disulfide bond	━	━
F420A	loop	✚	✚
S424A-S425A	loop	✚	✚
R432A	loop	✚	✚
R437A	loop	✚	✚
R432A-R437A	loop	✚	✚
H441R	η2	━	━
D444A	η2-β6	━	━

Mutation position represents the location of mutated residues in the simulated-structure of SRCR domain

✚, mutants are surface-targeted and do not internalize oAβ

━, mutants are not surface-targeted and do not internalize oAβ

Next, we assessed the role of SRCR domain in the protein trafficking of MARCO, another member of class I SRCR domain superfamily by expressing deletion variants with serial truncations of the SRCR domain in HEK293T cells. The truncated MARCO mutants 499, 476, 442, 431, and 420 contain 79, 56, 22, 11, and 0 residues of SRCR domain, respectively (Additional file 1: Figure S1A; Additional file 1: Table S4). The comparable enzymatic activities of co-transfected β-galactosidase across variants suggest that their transfection efficiencies were similar (data not shown). All of the truncated MARCO mutants were able to be expressed in HEK293T cells with the major molecular weight at approximately 60 to 50 kDa (Additional file 1: Figure S1B). However, only the mutant 442, 431 and 420 were surface-targeted (Additional file 1: Figure S1C).

To determine the critical residues of the MARCO SRCR domain for N-glycosylation and surface targeting of MARCO, and MARCO-mediated oAβ internalization, amino acid side chain inβ-sheet 1 (R425), β-sheet 2 (R432, G433, G434 and G436), β-sheet 3 (W443), α-helix (I475, V458, F459), disulfide bond (C460), β-sheet 4 (L471), η2 (H510A) and loop (L463, V486, D513) were site-directed mutated to hydrophobic alanine (A) (Fig. 4 and Additional file 1: Table S3). Western blot showed that all of the point mutants were able to be expressed in HEK293T cells with molecular weight at approximately 60 kDa (Fig. 6d). The expression levels of SRCR point mutants G433A, W443A, V458A, and F459A were lower than wild-type MARCO (Fig. 6d) although the transfection efficiency of all point mutants were similar (Data not shown) . The surface biotinylation assay and live immunostaining showed that R432A, L463A, L471A and D513A were surface-targeted (Fig. 6e and Table 3), and the oAβ internalization mediate by these mutants were not significantly changed (data not shown).

**Table 3:** Summary of the surface targeting of mutants in SRCR domain of human MARCO

Mutants	Mutation position	Surface targeting
R425A	β1	━
R432A	Loop-β2	✚
G433A	β2	━
R434A	β2	━
R436A	β2	━
W443A	β3	━
I457A	α1	✚
V458A	α1	━
F459A	α1	━
C460A	disulfide bond	━
L463A	loop	✚
L471A	β4-loop	✚
V486A	loop	━
H510A	η2	━
D513A	loop	✚

Mutation position represents the location of mutated residues in the simulated-structure of SRCR domain

✚, mutants are surface-targeted

━, mutants are not surface-targeted

## Discussion

In the present study, we use live immunostaining, surface biotinylation assay and FAM-labeled oAβ internalization to assess the function of N-glycosylation and the critical residuals of SRCR domain of Human SR-AI and MARCO. Human SR-AI is a transmembrane glycoprotein with seven predicted N-glycosylation sites [4]. However, utilization of the predicted N-glycosylation sites and their roles in surface targeting and functions of SR-AI remained unclear. The previous study has found that preventing N-glycosylation of SR-AI by inhibiting α-glucosidases I and II leads to retention of immature N-glycans on SR-AI, but the abilities of SR-AI surface targeting and ligand uptake are abolished [17]. In present study, we identified three N-glycosylation sites critical for the formation of complex-type N-glycans. The mutation of receptor’s N-glycosylation sites at N102Q, N143Q, N184Q, N102Q-N143Q, N102Q-N184Q and N143Q-N184Q significantly reduced receptor’s N-glycosylation without affecting its surface targeting. The level of oAβ internalization was diminished only as the mutation occurred at two successive N-glycosylation sites (i.e. N102Q-N143Q and N143Q-N184Q). Until now, the role of glycosylation status of scavenger receptor was only reported in two class B receptors. The structure and ligand binding of CD36 and SR-BI are modulated by the status of N-glycan. All 11 potential N-linked glycosylation sites were glycosylated in the murine SR-BI [31]. Mutations at two of these sites at positions 108 and 173 induced fully Endo H-sensitive forms of SR-BI and might have interfered with exit of the receptors from the ER, substantially reducing Golgi-mediated processing. Consequently, these two sites are critical for normal surface expression and efficient lipid uptake. Alternatively, site-directed mutagenesis of human class B scavenger receptor CD36 showed that mature N-linked glycosylation is not necessary for surface expression and ligand binding [32]. CD36 mutants with fewer glycosylation sites can fold, target to cell surface, and bind ligand. According to our data, double mutants N102Q-N184Q showed low expression level in membrane protein but is able to internalize as much ligand as wild-type SR-AI which was consistent with the study of CD36. Nevertheless, the N-glycosylation in double mutant N102Q-N143Q and N143Q-N184Q abrogated its activity on oAβ internalization.

The molecular weight of endogenous and surface protein of MARCO after PNGase F treatment was larger than the predicted molecular weight of MARCO. It is likely that MARCO contain other posttranslational modification. Because MARCO contains four predicted *O*-link glycosylation sites in collagenous structure (T189, T219, S326, and S329) and average molecular mass of single *O*-link polysaccharide is 1-2 kDa, it is possible that the shift of molecular size cause by *O*-glycosylation. The smear band spanning the molecular weight range of 55 to 70 kDa in the cell lysates were PNGase F sensitive, indicating that the MARCO exhibited different extents of *N*-link glycans. Moreover, *N*-glycosylation defected MARCO (i.e. N83Q-136Q double mutant) was not expressed on cell surface.

Our previous study has provided evidence that the intact SRCR domain of SR-AI is critical for the protein folding and N-glycosylation. The level of oAβ internalization was diminished in the cells expressing the surface targeted SRCR truncated variant 371 and 341 [4]. In the present study, the similar result was confirmed on MARCO. Disulfide bonds are important for protein folding and conformational stabilization as well as for protein maturation and trafficking [33]. The globular fold of SRCR domain of monomeric MARCO is stabilized by three disulfide bonds: C447-C508, C459-C517, and C487-C497 [14]. Truncated mutants 499 and 476 reduced the total MARCO expression which may be due to the decrease translation efficiency or increase protein degradation. Compared to truncated mutants 499 and 476, deletion the total cysteine residues mutants (442, 431, and 420) have higher level of MARCO. The data suggest that disulfide bonds of SRCR domain play a structural role for protein folding. Inhibition of disulfide bond formation of ATP-binding cassette sub-family G member 2 induced protein degradation in proteasomes [34]. In addition, the surface targeted mutant 442 has lower molecular weight than mutants 431 and 420, suggesting that the glycosylation pattern and polysaccharide length are variant among the three truncated mutants. It is possible that the process of *N*-glycosylation in MARCO was affected in mutant 442, since glycosylation is important for protein folding and stability, cell adhesion, and receptor targeting [16].

**Fig. 7:**
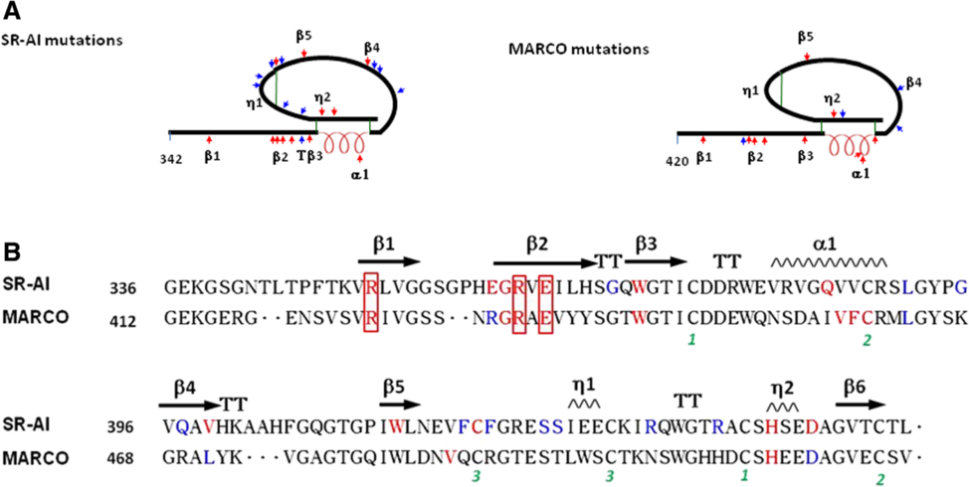
Comparison of the amino acid required for targeting surface and internalizing Aβ between SR-AI and MARCO. **a** Schematic representation of SRCR domain of SR-AI and MARCO and the mutation sites are shown. The surface targeted mutations are shown as blue arrows and the intracellular retained mutantions are shown as *red* arrows. The black line indicates the sequence of SRCR; α-helix is indicated as *red* spring, and disulfide bonds are shown as *green* line. The secondary structure of the SRCR domain of SR-AI, β-strands (β1-6), α-helix (α) and 3_10_ helices (η1, η2) are indicated. **b** Sequence alignment of human MACRO and SR-AI using ClustalW2 and ESPrip. The β-sheets are shown as arrows, and helixes are shown as a saw tooth pattern. Turns are marked as T. The three pairs of disulfide bonds are labeled as numbers in *green*. The amino acid in *red* indicates its mutation is not surface targetable. The amino acid in *blue* indicates its mutation is surface targetable and be able to internalize oAβ

We also provide the first evidence for that mutation of SRCR domain of SR-AI and MARCO at the residual in β sheets, α-helix and η2 and the cysteine residuals for disulfide bond formation abolished receptor’s trafficking. The similar clusters of acidic and basic residues in SRCR domain are similar between SR-AI and MARCO (Fig. 1b and c). One RRE motif, located at R351-R362-E364 in β-sheet 1 and 2 of SR-AI corresponds to R425-R454-E456 of human MARCO (Fig. 7). Three single point mutations at R351A and E364A of human SR-AI and that at R425A of human MARCO impaired receptor surface trafficking and N-glycosylation. This suggests that the SRCR domain of SR-AI may play a predominant role in protein folding. In a dimmer of the recombinant MARCO SRCR domain, β-sheet 1, the turn, and β-sheet 2 form a large β-sheet via β-strand swapping of the two monomers [14]. Our results demonstrated that all mutants at the region of β-sheet, α-helix and disulfide bound were retained intracellularly, suggesting that this region is critical for the protein folding and trafficking. Alternatively, all 9 mutations of SR-AI and 3 mutant of MARCO at the residual in turn or loops did not affect receptor’s surface trafficking and N-glycosylation. However, the receptor-mediated oAβ internalization was not significantly affected by those surface targeted mutations of SR-AI.

## Conclusions

In conclusion, our study identifies the critical motifs of SRCR domain regulating the proper folding required for surface trafficking which is prerequisite for exerting diverse functions of human MARCO and human SR-AI. Alternatively, N-glycan moiety is more critical than SRCR domain for SR-A-mediated oAβ internalization.

## Additional file

Additional file 1: Figure S1. The SRCR domain is critical for the surface targeting and N-glycosylation of MARCO. HEK293 cells were transfected with MARCO and variants with truncated SRCR domain. A. Constructs 499, 478, 442, 491 and 420 MARCO variants with truncated SRCR domains (blue line) and contact collagenous domain (red line). The secondary structure of the SRCR domain of SR-AI, β-strands (β1-6), α-helix (α) and 310 helices (h1, h2) are indicated in red. Disulfide bonds are shown in green. Total cell lysates (B) and avidin pull-down of biotinylated lysates (C) were subjected to Western blot analysis. The result was repeated for three times and the representative blot was shown. **Table S1:** Primer sets for expression constructs of SR-A1 mutants at spacer and coiled-coil domain. **Table S2:** Primer sets for expression constructs of SR-A1 mutants at SRCR domain. **Table S3:** Primer sets for expression constructs of MARCO point mutants. **Table S4:** The primer sets for expression construct of MARCO variants. (ZIP 962 kb)

## Abbreviations

Aβ: Amyloid-β peptide
AcLDL: Acetylated low-density lipoprotein
AD: Alzheimer’s disease
DMEM: Dulbecco’s modified Eagle’s medium
FAM: Fluorescein amidite
FBS: Fetal bovine serum
HEK293T: Human embryonic kidney cell line 293 T
SR-A: Scavenger receptor class A
SR-AI: SR-A type I
SR-BI: Scavenger receptor class B type I
SRCR: Scavenger receptor cysteine-rich domain
MARCO: Macrophage receptor with collagenous structure
oAβ: Oligomeric amyloid-β peptide
PNGase F: Peptide N-glycosidase
Endo H: Endoglycosidase
