# Supplementary material for: Identifying N-linked glycan moiety and motifs in the cysteine-rich domain critical for N-glycosylation and intracellular trafficking of SR-AI and MARCO
Source: J Biomed Sci. 2016 Feb 18;23:27. doi: 10.1186/s12929-016-0244-5 (PMC4758095; doi:10.1186/s12929-016-0244-5)
Supplement: Additional file 1: Figure S1. — The SRCR domain is critical for the surface targeting and N-glycosylation of MARCO. HEK293 cells were transfected with MARCO and variants with truncated SRCR domain. A. Constructs 499, 478, 442, 491 and 420 MARCO variants with truncated SRCR domains (blue line) and contact collagenous domain (red line). The secondary structure of the SRCR domain of SR-AI, β-strands (β1-6), α-helix (α) and 310 helices (h1, h2) are indicated in red. Disulfide bonds are shown in green. Total cell lysates (B) and avidin pull-down of biotinylated lysates (C) were subjected to Western blot analysis. The result was repeated for three times and the representative blot was shown. Table S1: Primer sets for expression constructs of SR-A1 mutants at spacer and coiled-coil domain. Table S2: Primer sets for expression constructs of SR-A1 mutants at SRCR domain. Table S3: Primer sets for expression constructs of MARCO point mutants. Table S4: The primer sets for expression construct of MARCO variants. (ZIP 962 kb) [file 12929_2016_244_MOESM1_ESM.zip › Additional file 1/Table S3.docx]

**Table S3. Primer sets for expression constructs of MARCO point mutants.**

| **MARCO mutant** | **primers** |  |
| --- | --- | --- |
| **R425A**  **R432A**  **G433A**  **R434A**  **R436A**  **W443A** | F  R  F  R  F  R  F  R  F  R  F  R | **AACTCAGTGTCCGTCGCAATTGTCGGCAGTAGT**  **ACTACTGCCGACAATTGCGACGGACACTGAGTT**  **GTCGGCAGTAGTAACGCAGGCCGGGCTGAAGTT**  **AACTTCAGCCCGGCCTGCGTTACTACTGCCGAC**  **GGCAGTAGTAACCGAGCACGGGCTGAAGTTTAC**  **GTAAACTTCAGCCCGTGCTCGGTTACTACTGCC**  **AGTAGTAACCGAGGCGCAGCTGAAGTTTACTAC**  **GTAGTAAACTTCAGCTGCGCCTCGGTTACTAC**  **AACCGAGGCCGGGCTGCAGTTTACTACAGTGGT**  **ACCACTGTAGTAAACTGCAGCCCGGCCTCGGTT**  **TACTACAGTGGTACCGCAGGGACAATTTGCGAT**  **ATCGCAAATTGTCCCTGCGGTACCACTGTAGTA** |
| **V458A** | F | **CTGATGCCATTGCCTTCTGCCGCATG** |
|  | R | **CATGCGGCAGAAGGCAATGGCATCAG** |
| **F459A** | F | **GATGCCATTGTCGCCTGCCGCATGC** |
|  | R | **GCATGCGGCAGGCGACAATGGCATC** |
| **C460A** | F | **CCATTGTCTTCGCCCGCATGCTGGG** |
|  | R | **CCCAGCATGCGGGCGAAGACAATGG** |
| **L463A** | F | **GTCTTCTGCCGCATGGCGGGTTACTCC** |
|  | R | **GGAGTAACCCGCCATGCGGCAGAAGAC** |
| **L471A** | F | **TCCAAAGGAAGGGCCGCATACAAAGTGGGAGCT** |
|  | R | **AGCTCCCACTTTGTATGCGGCCCTTCCTTTGGA** |
| **V486A** | F | **ATCTGGCTGGATAATGCACAGTGTCGGGGCACG** |
|  | R | **CGTGCCCCGACACTGTGCATTATCCAGCCAGAT** |
| **H510A** | F | **CATCATGACTGCAGCGCAGAGGAGGACGCAGGC** |
|  | R | **GCCTGCGTCCTCCTCTGCGCTGCAGTCATGATG** |
| **D513A** | F | **TGCAGCCACGAGGAGGCAGCAGGCGTGGAGTGC** |
|  | R | **GCCTGCGTCCTCCTCTGCGCTGCAGTCATGATG** |
